# Vacuum Phenomenon in the Lumbar Spine: Pilot Study for Accuracy of Magnetic Resonance Imaging

**DOI:** 10.5334/jbsr.3118

**Published:** 2023-11-02

**Authors:** Arvy Buttiens, Marian Simko, Johan Van Goethem

**Affiliations:** 1 Jessa Ziekenhuis, BE; 2 Medical imaging center Freiburg, DE; 3 VITAZ, BE

**Keywords:** MRI, gradient echo, sensitivity, vacuum phenomenon, lumbar spine

## Abstract

**Objectives:**

Vacuum phenomenon (VP) is defined as air within a joint. Many pathologies are associated with VP, mainly degenerative disease and trauma. Although patients with intradiscal gas may be asymptomatic, it promotes disc degeneration and can eventually become painful. VP is suspected to be an indicator of segmental mobility, helping in determining the extent of spinal fusion in a preoperative setting. This could make the detection of VP useful on routine magnetic resonance imaging (MRI) of the lower spine. We determined the accuracy of MRI in detecting intradiscal gas through a retrospective observational study.

**Materials and methods:**

The study population consists of 37 consecutive patients with low back pain who were scheduled for treatment with spinal infiltrations and received a computed tomography and MRI scan within a maximum time interval of 3 months. Spin echo (SE) T1 and T2 and gradient echo (GE) T1 sequences were analyzed. All scans were randomly coded and evaluated by two observers: an experienced neuroradiologist and a radiology resident for the presence of VP.

**Results:**

GE-imaging revealed a high accuracy with a sensitivity of 89.3%–92% and a specificity of 89.7–95.3% between both observers. In comparison to a sensitivity of 31.5%–76.3% for T1- and 8.5%–86.4% for T2-imaging and a specificity of 95%–100% for T1- and 63.7%–100% for T2-imaging with respective accuracy of 68.1%–85.7% and 54.6%–68.9%. We notice a moderate interobserver variability for the T1 (κ = 0.462) weighted imaging, no agreement for T2 (κ = 0.057) weighted imaging, and almost perfect interobserver variability for the GE sequence (κ = 0.889).

**Conclusion:**

The presence of VP in degenerative disc disease is a sign of segmental instability which is important for planning spinal fusion surgery. Our study showed that VP can be detected on MRI of the lumbar spine with high accuracy and almost perfect interobserver agreement by adding GE sequences to the scanning protocol.

## Introduction

Vacuum phenomenon (VP) is defined as air within a joint [2]. This phenomenon can be acute, caused by a rapid increase in joint volume, or chronic, due to degenerative changes [1]. VP has been described in multiple joints and is most commonly seen within the intervertebral discs, and will be referred to as intradiscal gas in the rest of this article [1,2]. It is usually reported as an incidental finding since it can be seen after normal joint motion [1,3]. However multiple pathologies have been associated with VP, mainly degenerative disease and trauma [1,2,3]. Although patients with intradiscal gas may be asymptomatic, it promotes disc degeneration and can eventually become painful [1]. VP is suspected to be an indicator of segmental mobility, helping in determining the extent of spinal fusion in a preoperative setting [1,4,5,6,7]. Thus, reporting VP on routine imaging can be of clinical importance, especially to help detect associated pathology. In this study, we determined the accuracy of magnetic resonance imaging (MRI) in detecting intradiscal gas compared to computed tomography (CT), which was used as the golden standard.

## Materials and methods

The study population consists of consecutive patients with low back pain who were scheduled for treatment with spinal infiltrations. All patients received a CT and MRI scan within a maximum time interval of 3 months. We included 37 patients in this study, 13 males and 24 females with ages ranging from 21 to 81 years. Exclusion criteria for spinal infiltration were neurological deficit, active infection, cancer-related pain, and <18 years old. Patients with known allergies to any of the medication that was used were premedicated with antihistamine and systemic corticosteroids for 48 h. General Electric’s Optima CT520 was used to perform the CT scans and General Electric’s Optima 450, 1.5T for the MRI scans. A total number of 125 lumbar discs were evaluated on CT; 8 at level L1-L2, 17 at level L2-L3, 26 at level L3-L4, and 37 at both level L4-L5 and L5–S1. The MRI scanning protocols consisted of sagittal and axial T2 spin echo (SE) with TR 2559–3297, TE 102–106, sagittal T1 gradient echo (GE) with TR 150, TE 4.2, axial proton density SE with TR 2310, TE 40, coronal T1 SE with TR 477–567, TE 13.94, and coronal neurography with myelography reconstructions. All scans were anonymized and evaluated blindly to avoid interpretation bias between the CT and MRI scans of the same patient. A random selection of 10 scans was scored twice at a different time, at least 2 months apart for the intraobserver variability. Afterwards, they were evaluated by two observers: a neuroradiologist with over 35 years of experience (observer 1) and a 3rd year radiology resident (observer 2) for the presence of VP in all separate lumbar discs from L1-L2 to L5-S1. Any transitional anomalies were noted by the observers to achieve the correct numbering of the intervertebral discs. Descriptive statistics were applied to the data using IBM SPSS Statistics 27 including kappa statistics to measure the intra- and interobserver variability.

## Results

The sensitivity and specificity for MRI to detect VP were determined, using the CT scans evaluated by observer 1 as golden standard. The sensitivity and specificity as well as positive predictive value (PPV), negative predictive value (NPV), and accuracy for the different MRI sequences—T1, T2, and GE—are shown for each observer in Table 1.

**Table 1:** Specificity and sensitivity.


		SENSITIVITY (%)	SPECIFICITY (%)	PPV (%)	NPV (%)	ACCURACY (%)

T1	Observer 1	31.5	100	100	61.2	68.1

	Observer 2	76.3	95	93.8	80.3	85.7

T2	Observer 1	8.5	100	100	52.6	54.6

	Observer 2	86.4	51.7	63.7	79.5	68.9

GE	Observer 1	86.7	95	94.5	87.7	90.8

	Observer 2	88.3	95	94.6	89.1	91.7


GE, gradient echo; NPV, negative predictive value; PPV, positive predictive value.

We notice no agreement to moderate interobserver variability for the T1- and T2-weighted imaging with observer 1 having perfect specificity (100%) at the cost of a very low sensitivity (31.5 and 8.5) on T1- and T2-weighted sequences, respectively. Observer 2 detected intradiscal gas on SE T1- and T2-weighted sequences with higher sensitivity (76.3 and 86.4, respectively) and with high specificity on T1 (95), but with low specificity on T2 (51.7). Overall, slightly higher accuracy was seen on T1-weighted images in both observers. Both observers did remarkably better in terms of sensitivity and specificity on GE sequences, resulting in high accuracy ratings for observer 1 (90.8) and observer 2 (91.7).

Interobserver agreement was moderate for T1 (0.462); none to slight agreement for T2 (0.057) and almost perfect for GE (0.889). For comparison, the interobserver variability for CT had a kappa value of 0.922. Intraobserver agreement was moderate for T1 (0.581–0.608); almost perfect for GE (0.866–0.910) and perfect for CT (1.00) in both observers, similar to the interobserver agreement. However, intraobserver agreement was not computable for senior observer 1 since all scans were negative for intradiscal gas during the second observation, in accordance with the low sensitivity of observer 1 (8.5% for T2). In contrast, moderate intraobserver agreement for T2 was found in observer 2 (0.567). All kappa values are summarized in Table 2.

**Table 2:** Kappa values of inter- and intraobserver variability.


VARIABILITY	T1	T2	GE	CT

**Interobserver**	0.462	0.057	0.889	0.922

**Intraobserver 1**	0.581	0.567	0.866	1

**Intraobserver 2**	0.608	N/A*	0.910	1


*No statistic computed since the second observation was a constant, all scans were scored as absent VP. CT, computed tomography; GE, gradient echo.

## Discussion

VP, also known as pneumoarthrosis, is defined by air or gas-like density—lower than approximately –300 HU—within a joint [1,2,8,9]. VP can be classified into acute, due to a rapid increase in the volume of a joint space and subacute or chronic seen with degenerative changes [1].

VP was first reported by Fick in his studies of joints under traction in the early 1900s [10]. Almost 30 years later Magnussen reported VP within the intervertebral disc and made the hypothesis that a significant reduction in pressure was needed to create the phenomenon [11]. Ten years later Knutsson associated VP with degenerative changes of the disc [12]. In 1977 Ford analyzed the content of intradiscal gas and found 90%–92% nitrogen [13].

The pathophysiology behind VP can be explained through biomechanics and physics. If a joint space expands, the volume increases and subsequently the pressure within the joint fluid will decrease (Boyle’s law). This decrease in pressure also decreases the solubility of gas (Henry’s law) and will result in the formation of gas within the joint. This mechanism explains the occurrence of an acute VP. How to explain the development of a chronic VP? However, Coulier hypothesized that a hydro-gaseous mixture can penetrate a partially permeable structure. This means that an elongation of a joint capsule will generate a decrease in pressure and allow fluids and gases to enter the joint from the adjacent tissues. When the joint is compressed or no longer elongated these fluids and gases can either reenter the adjacent tissues or form gaseous collections. Repetition of this mechanism will lead to VP, described as the pumping effect. Three different factors have been described to play a role in this process, the duration and depth of mechanical and metabolic alterations, the nature of neighboring tissues, and the permeability of the joint structures [1,9]. As the degenerative disc eventually becomes avascular and the endplate permeability drops due to progressive calcification, the accumulated gas within the disc cannot be cleared [1].

VP has been described in multiple joints, most commonly within the spine [2]. It is usually an incidental finding in patients scanned for other reasons [1]. It can be seen after normal joint motion, however multiple pathologies have been reported to be associated with VP [3]. These mainly include degenerative disease, trauma, osteoporotic fractures, and less commonly infectious diseases [1,2,3]. Patients with intradiscal gas may initially be asymptomatic, although VP alters biomechanical stress and promotes further disc degeneration [1]. This process can eventually lead to pain.

The literature on the prevalence of VP is rather limited. The prevalence of VP is around 2% within the general population and up to 20% in the elderly [13,14]. However, these numbers are based on conventional radiographs. Moreover, the detection of VP is influenced by patient positioning. Extension of the spine which is partially achieved in the supine position causes the intervertebral spaces to elongate slightly which causes expansion and increased visualization of intradiscal gas. Thanks to the high spatial resolution and good contrast resolution for gases on CT, it is the most sensitive imaging technique for VP [9]. Moreover CT scans are performed in the supine position which further increases the visualization of intradiscal gas. This results in a prevalence of 46% in the lumbar spines of patients older than 40 years [15].

MRI is widely used for imaging spinal pathology and is often the preferred imaging technique. However, protons are scarce in gas and will produce low or no signal. This makes it difficult to differentiate gas from calcifications, cortical bone, and reduced signal intensity in degenerative discs [1,16]. Moreover, the intrinsic noise of MRI might obscure the limited to absent signal from intradiscal gas. This leads to conventional MR imaging techniques being misleading with a low sensitivity for the detection of VP [2,16]. Some studies have reported that SE with low repetition time (TR) and echo time (TE), GE, and 3D sequences may be useful and even similar to CT for detecting VP, however [2,16,17]. Also increased TE in GE sequences increases susceptibility artifacts and can make gas more conspicuous [17].

Identification of VP is more difficult when located close to the endplates due to cortical bone having a similar low signal intensity and the presence of chemical shift artifacts at the endplates [16]. The chemical shift effect occurs inferiorly along the frequency encoding gradient, typically making the lower endplate appear thicker. A stronger magnetic field will increase this effect. Swapping the frequency and phase encoding direction can avoid this effect.

**Figure 1:**
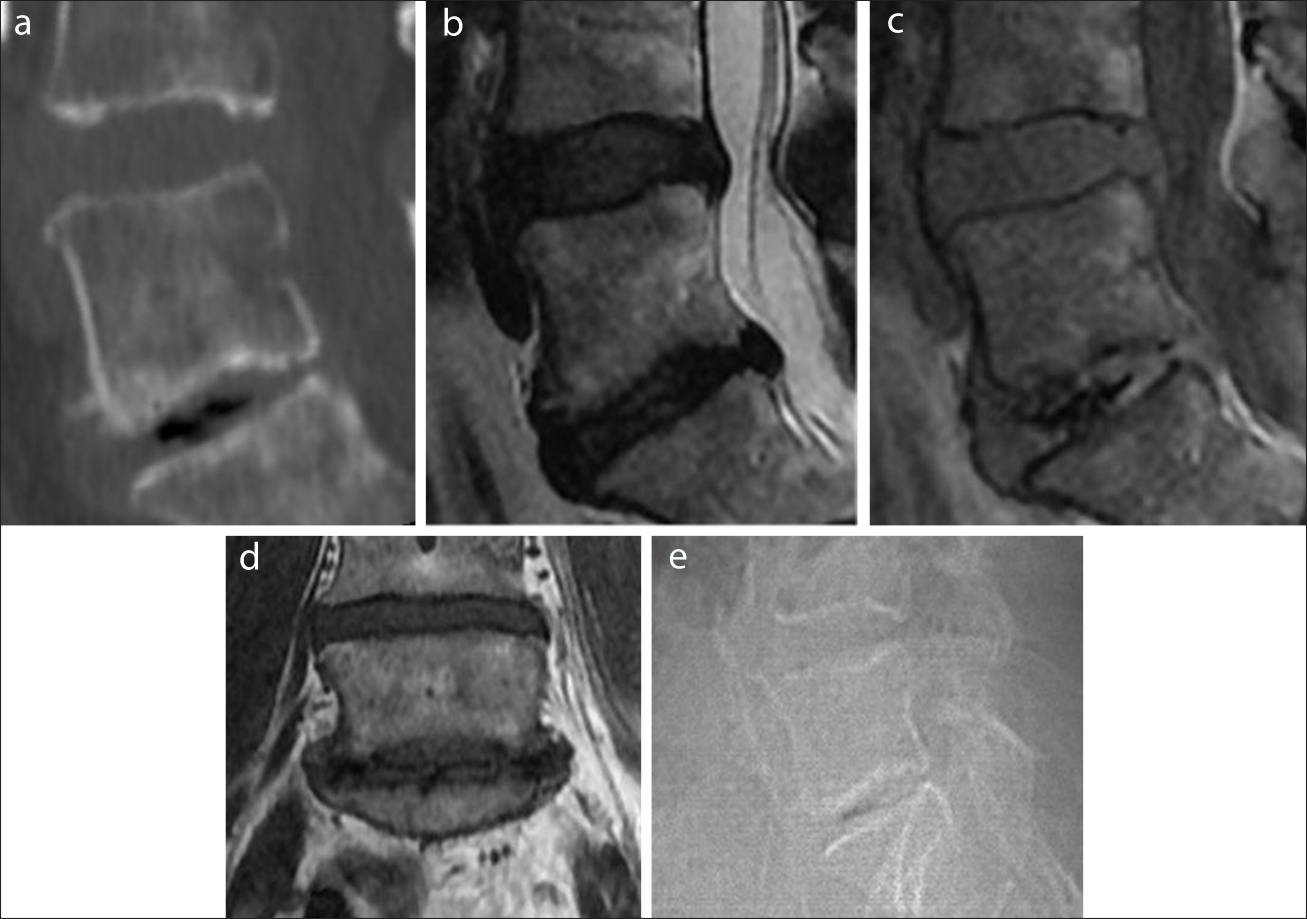
VP as intradiscal gas in the lower (L5-S1) of the two visualized discs (L4-L5 and L5-S1) on a sagittal reconstruction of a CT lumbar spine in bone window **(a)**, a sagittal T2- **(b)** and GE-weighted image **(c)**, a coronal T1-weighted image **(d)**, and the lateral scout image from the CT exam **(e)**.

Our study revealed high sensitivity (89.3%–92%) and specificity (89.7%–95.3%) on GE sequences with an almost perfect interobserver agreement (κ = 0.889). T2-weighted imaging did not appear to be useful in evaluating VP, with low accuracy and high interobserver variability. T1-weighted imaging performed better than T2-weighted imaging with high specificity and good PPV. See Figure 1 for an example of VP on the different imaging techniques in one of our study patients.

Since VP may be an asymptomatic finding and may resolve spontaneously, it generally does not require treatment. VP can be seen as an eventually painful marker of degenerative disk, however, and the latter should be the focus of therapeutic interventions [1].

There have been some reports of VP causing nerve root compression, due to a gas-filled disc herniation. In these cases, percutaneous aspiration of intradiscal gas can result in sudden relief of pain or improve symptoms and should be considered as first-line treatment. One patient included in our study had a gas-filled disc herniation that was percutaneously aspirated. Aspiration of VP does not treat the underlying mechanism and the recurrence rate is high [1,18].

Intradiscal gas is commonly regarded as a sign of segmental instability and may be an indication for fusion with interbody cages [7]. This instability is probably linked to the associated degeneration of the disc structure which has an important role in load distribution and provides flexibility to the spine [1,4,5,6,7].

This could make the detection of VP on routine MRI of the lower spine, which is frequently used for degenerative disc disease, useful for detecting associated pathologies and planning invasive spinal interventions [1,9]. It should be noted that conventional T1- and T2-weighted imaging are not reliable in the detection of VP and GE sequences should be incorporated in the scanning protocol when looking for the presence of VP.

Since this study only included a limited number of patients, our analysis can only be used as a pilot study for further investigations about the usefulness of MRI for detecting VP. Because of the limited previous literature on this specific subject, further investigation may prove useful and could confirm our preliminary results.

Analysis of VP on T1- and T2-weighted imaging revealed only moderate interobserver variability which would make it difficult to use in practice.

## Conclusion

The presence of VP in degenerative disc disease is a sign of segmental instability which is important for planning spinal fusion surgery. Our study showed that VP can be detected on MRI of the lumbar spine with high accuracy and almost perfect interobserver agreement be detected by adding GE sequences to the scanning protocol.
